# Assessing the Adaptive/Maladaptive Strategies of Mastication in Older Taiwanese Individuals Using the Masticatory Adaptation Experience Questionnaire (MAEQ)

**DOI:** 10.1111/joor.70181

**Published:** 2026-03-10

**Authors:** Chia‐Shu Lin, Ta‐Chung Chen, Yi‐Chen Chen, Li‐Jung Chao, Wei‐Chieh Kao, Jong‐Ling Fuh

**Affiliations:** ^1^ Department of Dentistry National Yang Ming Chiao Tung University Taipei Taiwan; ^2^ Institute of Brain Science National Yang Ming Chiao Tung University Taipei Taiwan; ^3^ Brain Research Center National Yang Ming Chiao Tung University Taipei Taiwan; ^4^ Oral Medicine Innovation Center National Yang Ming Chiao Tung University Taipei Taiwan; ^5^ Division of Prosthodontics, Department of Stomatology Taipei Veterans General Hospital Taipei Taiwan; ^6^ Department of Neurology, Neurological Institute Taipei Veterans General Hospital Taipei Taiwan; ^7^ School of Medicine, College of Medicine National Yang Ming Chiao Tung University Taipei Taiwan

**Keywords:** adaptation, aging, cognitive impairment, eating, food, mastication

## Abstract

**Background:**

There are various tests to assess the individual performance of mastication. However, it has remained unknown how individuals used adaptive (e.g., ‘increasing time to chew’) and maladaptive (e.g., ‘avoiding eating the food’) strategies to chew and whether aging/cognitive impairment plays a key role in the use of these strategies during eating.

**Objective:**

The study aims to develop the Masticatory Adaptation Experience Questionnaire (MAEQ) and investigate age‐related factors of masticatory adaptation.

**Methods:**

The study consists of two subsets: Set 1 included 125 healthy younger (YA, 20–50 years) and older (OA, over 50 years) adults, and Set 2 included 40 older patients with cognitive impairment (CI). Based on Set 1, internal consistency, test–retest reliability, and content and criterion‐related validity were assessed. Eating difficulty was assessed using a food‐specific questionnaire. The MAEQ mean score and scores for each adaptive/maladaptive strategy were compared between YAs and OAs, as well as between CIs and OAs.

**Results:**

The MAEQ, a questionnaire assessing the frequency of using adaptive/maladaptive strategies to eat, shows good reliability and validity in older adults. Compared to young adults, older adults used multiple adaptive and maladaptive strategies concurrently when they had greater eating difficulty. In contrast, cognitively‐impaired patients maladaptively avoid eating foods when having greater eating difficulty.

**Conclusion:**

The MAEQ is a valid tool for assessing the use of adaptive/maladaptive strategies during mastication in older individuals. Age and cognitive impairment may play a key role in the use of the strategies.

## Graphical abstract

The study aims to establish the Masticatory Adaptation Experience Questionnaire (MAEQ) and investigate age‐related factors of masticatory adaptation.

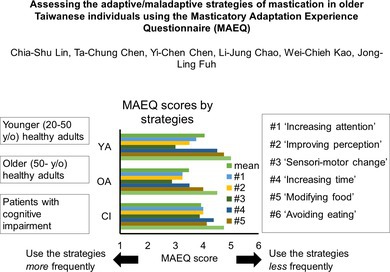

## 1 Introduction

Masticatory function is pivotal to eating and nutrient intake, and strongly associated with systemic health [1, 2]. For example, older individuals with tooth loss showed a higher risk of malnutrition [3], and a lower ability to chew hard food was associated with a higher risk of malnutrition [4]. At present, there have been various objective tests and questionnaires developed to assess individual masticatory performance and subjective eating experience, respectively [5]. Systematic reviews have confirmed that a lower masticatory performance, which is assessed via standardised tests, is associated with multiple oral health factors, including the number of missing teeth and oral muscular strength [6, 7].

However, the associations between masticatory performance (via objective tests) and subjective experience of eating difficulty (via questionnaires) were not consistent in the literature [8, 9]. Patients receiving implant‐supported prosthesis showed a poor association between masticatory performance and subjective experience of masticatory function [8]. In older individuals, while subjective experience of food intake was associated with the number of missing teeth, masticatory performance was associated with multiple factors (e.g., muscle tone) in addition to tooth loss [9]. Moreover, earlier research has shown that older individuals with no occlusal support (i.e., Eichner class C) can still preserve half of the masticatory performance, compared to individuals of Eichner class A [10]. The findings suggest that older individuals, even with a suboptimal oral condition (e.g., more missing teeth and lower masticatory performance), still preserve part of the masticatory function and maintain nutrient intake.

Previous studies have suggested that behavioural adaptation—using different approaches to cope with the suboptimal oral conditions—may play a key role in eating and nutrient intake. For example, older individuals with more missing teeth may increase the cycle of mastication before swallowing a food bolus [11, 12], pay more attention to the bolus during chewing, change different teeth to chew [13], or modify the texture of food (e.g., making it softer) [13, 14]. Individuals may also adopt a maladaptive strategy, for example, avoid eating some foods that they have difficulty chewing [11, 13]. In general, older individuals may subjectively perceive an adequate ability to eat foods by using multiple adaptive strategies to compensate for their poor oral conditions. To this general hypothesis, there are several key questions that remain untested:

- What are the most common strategies for masticatory adaptation during eating? Using questionnaires, previous studies showed that older individuals had great difficulty in eating some foods in their daily meals [15, 16]. Findings from qualitative research suggest that individuals with missing teeth would adopt different adaptive or maladaptive strategies to chew and maintain eating [13]. Until now, there has been no quantitative data reported for the frequency of using these adaptive or maladaptive strategies.
- Is the use of adaptive/maladaptive strategies associated with age‐related factors? Aging is associated with sensorimotor learning and adaptation [17], including the adaptation of masticatory functions [18, 19]. As age increased, older individuals showed a lower proportion of successful accommodation (i.e., showing no reduced activities with the help of a device) [20]. Notably, during mastication, many adaptive strategies are engaged with cognitive functions, for example, paying more attention to the size and texture of the food bolus. It is unclear if aging and cognitive impairment play a key role in the subjective experience of masticatory adaptation during eating.

The current study aims to establish a quantitative assessment, the Masticatory Adaptation Experience Questionnaire (MAEQ), for quantifying the frequency of using adaptive/maladaptive strategies during mastication in adults, and to investigate the factors associated with masticatory adaptation. The study aims to test the following three hypotheses:

Hypothesis 1 *Based on the data from healthy adults, the MAEQ shows good internal consistency, temporal stability, content validity, and criterion‐related validity with individual ratings of eating difficulty as the criterion (i.e., greater eating difficulty, a higher frequency to use adaptive/maladaptive strategies during mastication)*.

Hypothesis 2 *The MAEQ scores are lower in older, compared to younger adults, suggesting a higher frequency of using adaptive/maladaptive strategies in older individuals*.

Hypothesis 3 *The MAEQ scores are higher in older individuals with cognitive impairment, compared to older healthy adults, suggesting the association between cognitive impairment and the use of adaptive/maladaptive strategies*.

## 2 Materials and Methods

### 2.1 Study Design

The current study consists of two study sets of cross‐sectional data. Study Set 1 consists of younger healthy adults (YA) and older healthy adults (OA). This study set was used to establish the psychometric properties of the MAEQ and to investigate age as a factor of masticatory adaptation. Study Set 2 consists of older patients with cognitive impairment (CI). This study set was used to investigate cognitive impairment as a factor of masticatory adaptation by contrasting against the OA group from Study Set 1.

### 2.2 Participants

Study Set 1 consists of 125 healthy controls aged between 20 and 84 years (see Table 1 for inclusion and exclusion criteria for participant recruitment). Study Set 2 consists of 40 patients with CI, including patients with mild cognitive impairment (MCI) or Alzheimer's disease (AD), aged between 50 and 83 years. Study Set 1 was recruited via the advertisement announced in the campus of National Yang Ming Chiao Tung University (NYCU) and local community facilities. The participants were further divided into the YA (aged < 50 years) and the OA (aged ≧ 50 years) groups. The cut‐off age of 50 years old was used because in older individuals, major systemic diseases, such as cardiovascular diseases and cognitive decline, can be preceded by predisposing factors at a younger age and therefore investigated from 50 years old [21]. In terms of nutrition and eating behaviour, the cut‐off at 50 years old has been used in large‐sample cohort studies and meta‐analysis on dietary behaviour, obesity, and physical frailty [22, 23, 24]. The cut‐off age was also in parallel with Study Set 2, in which CI patients may have an early onset from 50 years old [25]. Study Set 2 was recruited via the outpatient departments of the Department of Neurology from Taipei Veterans General Hospital (VGHTPE) by a neurologist, according to the established clinical criteria [26, 27]. The diagnosis of amnesic MCI was made according to the revised consensus criteria from 2004 [26] which required the following conditions: (a) subjective cognitive complaint, (b) objective cognitive impairment in one or more cognitive domains (typically defined as performance 1 to 1.5 standard deviations below age‐ and education‐adjusted norms), and (c) preserved basic activities of daily living and not demented [26]. The diagnosis of Alzheimer's dementia was made according to the following criteria [27]: (a) decline from previous level, (b) interference with daily function, not due to delirium or major psychiatric illness, and (c) impairment in ≥ 2 cognitive domains confirmed by history and testing. Probable AD dementia requires insidious onset and progressive worsening, amnestic or non‐amnestic presentation (language, visuospatial, or executive) and no evidence of substantial cerebrovascular disease, Lewy body features, frontotemporal dementia, or other causes. Notably, there are overlaps in the research participants between Study Set 1 and previously published studies [18, 19] and between Study Set 2 and a previous study [28]. All the patients were screened by the neurologist to ensure that they were able to communicate verbally and were able to understand the questionnaire.

**TABLE 1:** The inclusion and exclusion criteria of study subsets.

	Study Set 1 (healthy adults)	Study Set 2 (cognitive impairment)
Site	National Yang Ming Chiao Tung University (YM109057F)	Taipei Veterans General Hospital (2021‐08‐004CC)
Inclusion criteria	able to independently communicate with the researchers and provide written informed consent, and aged between 20 and 85 years^a^	aged between 50 and 85, and being able to voluntarily participate in this study and conduct informed consent
Exclusion criteria	a medical history of temporomandibular disorders (TMDs) or showing symptoms of TMD, and acute dental or orofacial pain at the time of the study^a^	not able to perform the informed consent procedure

^a^ The inclusion and exclusion criteria were the same as the previously published study [18] for the participants were recruited under the same research project.

All the participants have signed a written informed consent before the research began. Study Set 1 was approved by the Institutional Review Board (IRB) of the NYUC (Code: YM109057F), and Study Set 2 was approved by the IRB of the VGHTPE (Code: 2021‐08‐004CC).

### 2.3 MAEQ Assessment

#### 2.3.1 Construction of the MAEQ

First, the adaptive and maladaptive strategies of mastication were searched from the literature, and potential items were included in the original draft of the questionnaire, which consisted of four adaptive strategies and one maladaptive strategy (Table 2). The original draft was revised by three experts who specialise in prosthodontics or nursing. The content validity of each strategy and the clarity of its description were assessed independently by the experts, with a numerical rating scale from 0 (not relevant/not clear at all) to 5 (very relevant/very clear). Based on their opinions, a new strategy (Table 2) was included to form the final version of the MAEQ (see Table S1 for the final version in Chinese).

**TABLE 2:** The adaptive and maladaptive strategies listed in the MAEQ.

	Description	Mechanism	Adaptation	Content validity	
				Clarity	Relevance
(A) Strategy					
#1	I pay much attention to whether my teeth (or dentures) break or crush the food well	Increasing attention	Adaptive	4.7	5.0
#2	I focus on perceiving the size, hardness, and texture of the food (or bolus) in my mouth, such as how elastic or how sticky it is	Improving perception	Adaptive	4.3	4.7
#3	I change to the different side or areas of my teeth, or other parts of the oral cavity (such as the gums), to break or crush the food	Sensori‐motor change	Adaptive	4.3	5.0
#4	I try to spend more time or apply more force when chewing it	Increasing time	Adaptive	4.7	5.0
#5	I change the way to cook or prepare the food (e.g., by steaming or cutting it into smaller pieces) because it is not easy to eat	Modifying food	Adaptive	*	*
#6	I avoid eating this kind of food because it is too difficult for me to break or crush it	Avoiding eating	Maladaptive	4.0	5.0
(B) Food					
#1	Sliced orange				
#2	Sliced guava				
#3	Stirred peanuts				
#4	Fried chicken fillets				

*Note:* *An additional question included according to the comment of the experts.

#### 2.3.2 Organisation of the MAEQ

The MAEQ aims to assess the frequency with which individuals have used these strategies in the past 3 months. The questionnaire consists of five descriptions of adaptive strategies (i.e., #1‐#5 in Table 2A) and one description of a maladaptive strategy (i.e., #6 in Table 2A). Because individual eating behaviour may change depending on food types, the six descriptions were assessed separately for four foods commonly seen in Taiwan, that is, sliced orange, sliced guava, stir‐fried peanuts, and fried chicken fillets, based on a previous survey on Taiwanese participants (Table 2B) [15]. Therefore, the whole MAEQ consists of 24 descriptions. The participants were instructed to rate the frequency of adopting each adaptive/maladaptive strategy and for each food, using a 1–5 numerical scale (1 = always and 5 = never). To quantify the masticatory adaptation, three types of scores were calculated:

- The MAEQ mean score: the mean value across 24 descriptions, that is, a general index for all strategies and all foods. A lower MAEQ score denotes a greater frequency of using adaptive/maladaptive strategies during mastication.
- The MAEQ score for each strategy: the mean score for one specific strategy averaged from all four foods.
- The MAEQ score for each food: the mean score for one specific food averaged from all six strategies.

#### 2.3.3 Procedure of the Assessment

In Study Set 1, the MAEQ was used as a self‐report assessment and completed by the participants individually. In addition, to assess the test–retest reliability of the MAEQ, 19 participants were additionally recruited to conduct the assessment twice with an interval of 4 weeks. All the participants for the test and retest were recruited based on the same criteria as Study Set 1. In Study Set 2, the MAEQ was completed by the patients with CI under the assistance of a research assistant, if needed, to ensure that the patients understood the items and gave a valid response. Each participant has to respond to the six questions (descriptions of masticatory experience) for each type of food, and give the responses to four types of food individually. Therefore, the whole questionnaire consists of 24 questions.

### 2.4 Clinical Assessments

In Study Set 1 and Study Set 2, eating difficulty was assessed using the food intake questionnaire [15]. The questionnaire consists of 23 common Taiwanese foods. The participants were asked to rate their subjective experience of eating the food (0: easy to chew, 1: difficult to chew, and 2: impossible to chew) for the 23 foods. Among the 23 foods, eight were related to fruits, five related to meats, and 10 related to vegetables. Eating difficulty was quantified by the mean score across all 23 foods and for each food type (fruits, meats, and vegetables). The higher score represents greater eating difficulty.

Additionally, in Study Set 1, six clinical factors of oral health and function were assessed for both YA and OA groups, based on the published protocols [29]:

- The number of missing teeth: The residual roots that were not functional during mastication were considered missing teeth. Fixed prostheses, including an implant‐supported crown or a fixed partial denture, were not considered missing teeth.
- Masticatory performance of food‐mixing: the participants chewed a fruit‐chew for 20 strokes, and the degree of colour mixing was quantified for their food‐mixing ability.
- Stimulated salivary flow rate: the participants chewed an odourless gum for 3 min, and the saliva was collected to estimate its flow rate.
- Maximal tongue pressure: tongue pressure was measured as the isometric force applied on an intraoral bulb placed in the posterior tongue.
- Maximal grip force: maximal grip force was assessed using a handheld dynamometer.
- Repetitive saliva swallowing test: The number of successful swallows within 30 s was recorded.

It is noteworthy that Study Set 1 was collected as part of a project on oral and brain functions [19, 29], and therefore, the results of these assessments were presented in these previous publications [19, 29]. In the current study, the assessment scores were used as the variables for clarifying the association between the MAEQ score and eating difficulty (for detailed procedures, please see 2.5.5).

### 2.5 Statistical Analyses

#### 2.5.1 Descriptive Analysis

Descriptive analyses were conducted by summarising the mean, median, standard deviation, interquartile range, minimum, and maximum, across the research variables: age, the number of missing teeth, the MAEQ mean score, the MAEQ score for each strategy, the MAEQ score for each food, eating difficulty (for all foods) and eating difficulty for fruits, meats, and vegetables. The analysis was conducted for the YA, OA, and CI groups, respectively. Normality of the distribution of all scores was assessed using the Shapiro–Wilk test.

#### 2.5.2 Analyses of Hypothesis 1

In terms of the reliability of the MAEQ, internal consistency of the 24 descriptions was quantified by Cronbach's alpha, with a value > 0.7 indicating an acceptable internal consistency. Test–retest reliability was assessed by intraclass correlation coefficient (ICC) with a two‐way mixed model, for the fixed effect of test–retest interval and the random effect of participants, and single measurement. The absolute agreement estimated by the ICC is expected to be over 0.75, which indicates good reliability [30]. The Bland–Altman plot was used to investigate the agreement of test and retest scores across participants.

In terms of the validity of the MAEQ, content validity was assessed by the three experts for the relevance and the clarity of the descriptions (see **2.3.1**). Criterion‐related validity was assessed using the ratings of eating difficulty (for all food types) as the criteria. The association between the MAEQ mean score and eating difficulty was assessed using analyses of partial correlation, controlled for age, sex, and number of missing teeth. The analyses were conducted for all the participants in Study Set 1, and respectively for the YA and OA groups.

#### 2.5.3 Analyses of Hypothesis 2

The YA and OA groups from Study Set 1 were compared for the MAEQ mean score, the MAEQ score for each strategy, and the MAEQ score for each food. The ratings of eating difficulty for all food types and for each food type were also compared between the age groups. The comparison was conducted using the Mann–Whitney *U* test because the scores were mostly non‐normally distributed (Table 3).

**TABLE 3:** Results of descriptive analyses on research variables.

Clinical	Mean	Median	Std	Min	Max	IQR	SW test
Age							
YA	27.7	25.0	6.4	20.0	47.0	10.0	0.000
OA	68.2	69.0	8.4	52.0	84.0	12.3	0.136
CI	72.0	74.5	8.4	50.0	83.0	11.8	0.005
Number of missing teeth							
YA	0.4	0.0	0.9	0	4	0.0	0.000
OA	3.3	1.0	5.7	0	28	4.0	0.000

MAEQ	Mean	Median	Std	Min	Max	IQR	SW test
Mean score							
YA	3.9	4.1	0.8	2.2	5.0	1.5	0.008
OA	3.5	3.5	0.9	1.0	5.0	1.4	0.204
CI	3.8	3.9	0.8	1.5	4.9	1.1	0.027
Strategy #1							
YA	3.5	3.8	1.3	1.0	5.0	2.0	0.001
OA	3.2	3.3	1.2	1.0	5.0	2.1	0.003
CI	3.5	4.0	1.2	1.0	5.0	1.7	0.001
Strategy #2							
YA	3.4	3.5	1.3	1.0	5.0	2.3	0.003
OA	3.2	3.3	1.2	1.0	5.0	1.8	0.005
CI	3.6	4.0	1.0	1.0	5.0	1.2	0.000
Strategy #3							
YA	3.1	3.0	1.3	1.0	5.0	2.3	0.013
OA	2.9	2.9	1.2	1.0	5.0	2.0	0.005
CI	3.4	3.9	1.4	1.0	5.0	2.3	0.001
Strategy #4							
YA	4.0	4.5	1.0	1.8	5.0	1.8	0.000
OA	3.5	3.5	1.1	1.0	5.0	1.8	0.005
CI	4.0	4.4	1.1	1.0	5.0	1.4	0.000
Strategy #5							
YA	3.5	4.8	2.1	0.0	5.0	2.5	0.000
OA	3.9	4.0	1.3	0.0	5.0	2.0	0.000
CI	3.9	4.1	1.0	1.8	5.0	1.7	0.001
Strategy #6							
YA	4.8	5.0	0.6	1.0	5.0	0.0	0.000
OA	4.2	4.5	1.0	1.0	5.0	1.3	0.000
CI	4.3	4.8	1.0	1.0	5.0	1.2	0.000
Food #1							
YA	3.9	4.2	0.9	2.0	5.0	1.5	0.001
OA	3.5	3.5	0.9	1.0	5.0	1.4	0.117
CI	3.8	3.8	0.9	1.7	5.0	1.3	0.026
Food #2							
YA	3.7	3.8	0.9	2.2	5.0	1.5	0.013
OA	3.4	3.5	1.0	1.0	5.0	1.7	0.033
CI	3.7	4.0	1.0	1.0	5.0	1.5	0.001
Food #3							
YA	4.0	4.2	1.0	1.5	5.0	1.5	0.000
OA	3.5	3.6	1.0	1.0	5.0	1.5	0.018
CI	3.9	4.0	0.8	2.2	5.0	1.1	0.027
Food #4							
YA	3.9	3.8	0.9	1.2	5.0	1.5	0.008
OA	3.5	3.5	1.1	1.0	5.0	1.5	0.012
CI	3.8	4.0	1.0	1.0	5.0	1.6	0.014

Eating difficulty	Mean	Median	Std	Min	Max	IQR	SW test
Mean score							
YA	0.06	0.04	0.06	0.00	0.22	0.09	0.000
OA	0.12	0.09	0.19	0.00	1.05	0.14	0.000
CI	0.17	0.09	0.25	0.00	1.22	0.22	0.000
Fruits							
YA	0.09	0.13	0.08	0.00	0.25	0.13	0.000
OA	0.15	0.13	0.20	0.00	1.00	0.25	0.000
CI	0.21	0.13	0.23	0.00	1.13	0.38	0.000
Meats							
YA	0.09	0.00	0.16	0.00	0.60	0.20	0.000
OA	0.22	0.00	0.30	0.00	1.20	0.40	0.000
CI	0.31	0.00	0.49	0.00	1.60	0.60	0.000
Vegetables							
YA	0.02	0.00	0.04	0.00	0.20	0.00	0.000
OA	0.05	0.00	0.19	0.00	1.22	0.00	0.000
CI	0.08	0.00	0.24	0.00	1.40	0.08	0.000

Abbreviations: CI, patients with cognitive impairment; OA, older healthy adults; YA, younger healthy adults.

Furthermore, two additional analyses were conducted to investigate the cross‐correlation between the research variables. First, cross‐correlation was investigated for each pair of strategies, which showed whether participants used two strategies concurrently. Second, the correlation between each of the strategies and the eating difficulty of each food type (fruits, meats, and vegetables) was investigated to clarify if a strategy was specifically for a type of food. Analyses of partial correlation were conducted by including age, sex, and number of missing teeth as the controlled variables. The analyses were conducted respectively for the YA and OA groups.

#### 2.5.4 Analyses of Hypothesis 3

Among the OA group (*n* = 74) in Study Set 1, 40 were selected to match the CI patients from Study Set 2 for their age and sex. Next, the OA subgroup (*n* = 40) and the CI group were compared for the MAEQ mean score, the MAEQ score for each strategy, and the MAEQ score for each food, using the Mann–Whitney *U* test. Notably, eating difficulty was not independently investigated because the findings have been reported in an earlier study [28]. Cross‐correlation of the MAEQ score of each strategy, as well as the association between MAEQ scores and eating difficulty, were investigated with the same analyses in Section 2.5.3.

#### 2.5.5 Predictive Models of Eating Difficulty

In addition to the analyses of the major hypotheses, regression analyses were conducted to elucidate the association between the MAEQ scores and eating difficulty. Two regression models were established. In the OA group, the first model (‘MAEQ mean score’) included the MAEQ mean score, age, sex, and the following six clinical variables of oral health and function: the number of missing teeth, masticatory performance, stimulated salivary flow rate, maximal tongue pressure, maximal grip force (as an indicator of physical strength), and the number of repetitive saliva swallowing, as the predictors and eating difficulty for all food types as the dependent variable. In the CI group, the model included only the MAEQ mean score, age, and sex, as the predictors. These models investigated whether masticatory adaptation is predictive of individual differences in eating difficulty. The second model (‘Multiple strategies’) included the predictors with statistical significance in the first model and replaced the MAEQ mean score with the scores of the six strategies. The model investigated which strategies are predictive of eating difficulty. In all the regression analyses, the variance inflation factor (VIF) of each variable and the Durbin‐Watson (DW) test were conducted to assess the risk of multicollinearity and autocorrelation.

The significance level for all the statistical tests was set at 0.05. All the statistical analyses were conducted using IBM SPSS Statistics (version 24).

## 3 Results

### 3.1 Descriptive Analysis

The descriptive data of the research variables are summarised in Table 3, including the MAEQ average score, the scores for each of the six strategies, the scores for each of the four foods, and eating difficulty for all foods, fruits, meats, and vegetables, respectively. The data are separately presented for the YA group, the OA groups (Study Set 1), and CI patients (Study Set 2). Notably, for the majority of research variables, the scores showed a non‐normal distribution, judging from the results of the Shapiro–Wilk test (i.e., the null hypothesis not being rejected at *p* = 0.1). Therefore, in the following analyses of hypothesis testing, non‐parametric methods were used.

**FIGURE 1:**
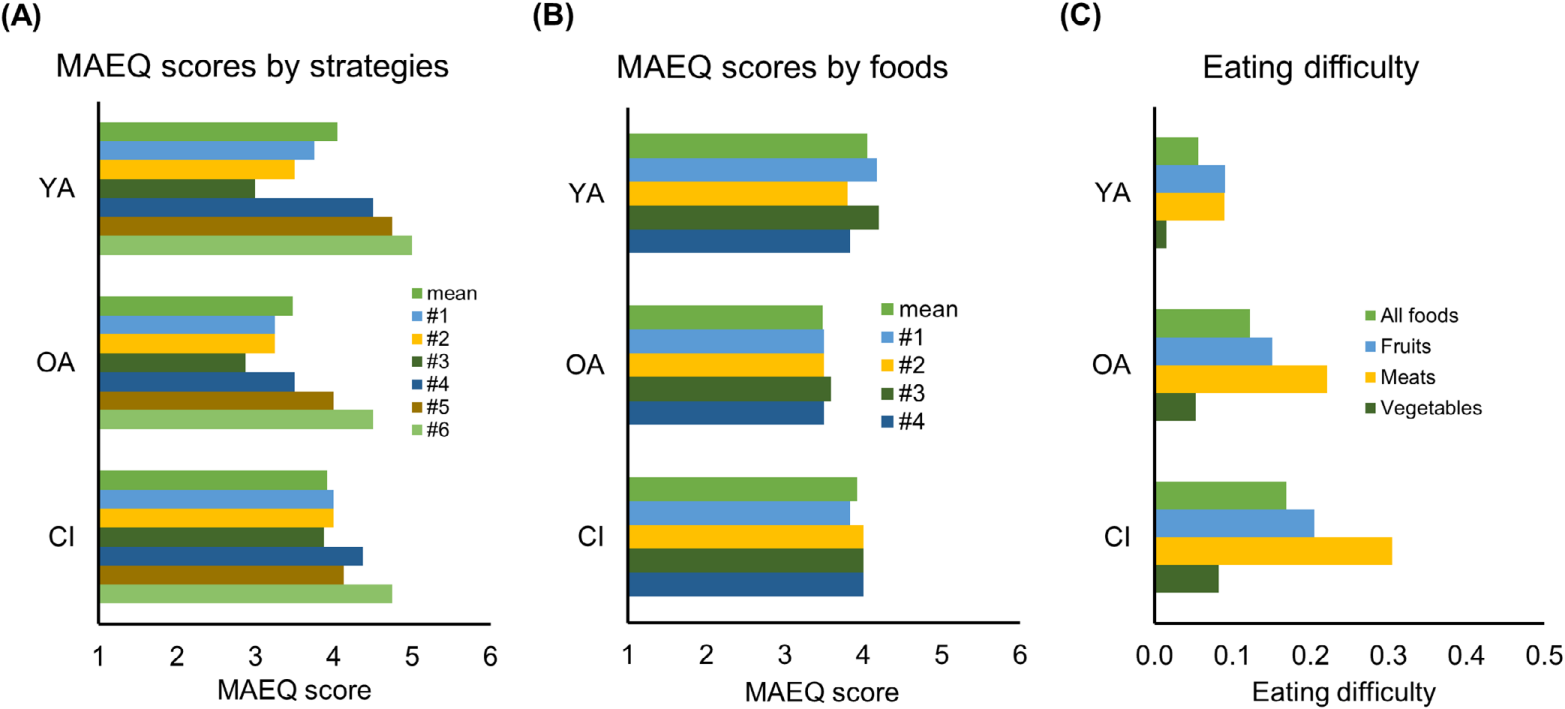
The frequency of using adaptive/maladaptive strategies for specific strategies (A), for specific foods (B), and eating difficulty (C) in younger healthy adults (YA), older healthy adults (OA), and patients with cognitive impairment (CI).

As shown in Figure 1A, the three groups showed different patterns of using MAEQ strategies. In both the YA and the OA groups, the strategy #3 ‘Sensorimotor change’ was more often adopted, compared to other strategies. However, CI patients showed a more even pattern, that is, the frequencies of using the adaptive strategies (#1 to #5) were similar to each other. For all the subgroups, the maladaptive strategy (#6 ‘Avoiding eating’) was used the least often. In contrast, for each group, the frequencies of using adaptive/maladaptive strategies were similar for different foods (Figure 1B). Finally, both the OA group and CI patients have the greatest difficulty in eating meats (Figure 1C).

### 3.2 Results of Hypothesis 1—Psychometric Features of the MAEQ

#### 3.2.1 Reliability of the MAEQ

**FIGURE 2:**
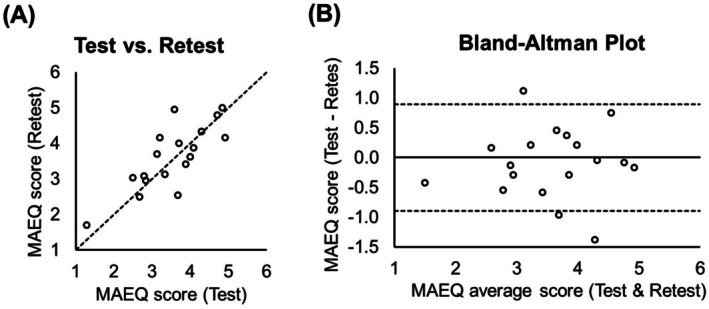
(A) The *x*–*y* plot depicting the association between test and retest scores of the MAEQ by a duration of one month. (B) The Bland–Altman plot. The dashed line in the Bland–Altman plot denotes the range of 1.5 standard deviations.

Based on the findings from Study Set 1, the MAEQ showed good internal consistency for the YA group (Cronbach's alpha = 0.945, *n* = 51) and the OA group (Cronbach's alpha = 0.960, *n* = 74). The absolute agreement estimated by the ICC = 0.79 (two‐way mixed model), showing an acceptable test–retest reliability (Figure 2A). The Bland–Altman plot showed a homogenous distribution of the testing scores across individual participants (Figure 2B). The findings support Hypothesis 1 that MAEQ presents good internal consistency and temporal stability for a duration of 1 month.

#### 3.2.2 Validity of the MAEQ

We first examined the content validity assessed by the expert panels. For the original draft of the MAEQ, the average rating on the ‘clarity’ of the questionnaire was 4.4 (4.0–4.7), and the average rating on its ‘relevance’ was 4.9 (4.7–5.0). According to the opinions of the experts, an additional question (i.e., #5 in Table 2) was added. Second, we assessed the criterion‐related validity using the score of eating difficulty as the criterion. Based on Study Set 1, we found that a higher frequency of using adaptive/maladaptive strategies was significantly associated with a higher degree of eating difficulty in all participants (*ρ* = −0.33, *p* < 0.001, *n* = 125). The correlation was statistically significant in the OA group (*ρ* = −0.39, *p* = 0.001, *n* = 74) but nonsignificant in the YA group (*ρ* = −0.16, *p* = 0.30, *n* = 51). The findings suggest a good clinical validity of the MAEQ for older but not younger individuals.

In addition, the previous study revealed that patients with temporomandibular disorders (TMDs) and muscular pain showed a significantly lower MAEQ score, compared to non‐TMD participants [31]. The findings provided additional evidence of the construct validity of the MAEQ that the score can discriminate between the clinical groups with and without deficits in mastication [31]. In general, the findings support Hypothesis 1 that the MAEQ presents good clinical validity when it is used for older adults.

### 3.3 Results of Hypothesis 2—Aging and Masticatory Adaptation

The OA group showed a significantly lower MAEQ mean score (Mann–Whitney *U* test, two‐tailed *p* = 0.019), score of strategy #4 (Mann–Whitney *U* test, two‐tailed *p* = 0.004), and score of strategy #6 (Mann–Whitney *U* test, two‐tailed *p* < 0.001), compared to the YA group. The OA group also showed a significantly lower MAEQ score in food type #1 ‘orange’ (Mann–Whitney *U* test, two‐tailed *p* = 0.007) and #3 ‘stirred peanuts’ (Mann–Whitney *U* test, two‐tailed *p* = 0.016), compared to the YA group, and a trend of statistical significance in food type #2 ‘guava’ (*p* = 0.06) and #4 ‘fried chicken’ (*p* = 0.08). Finally, the OA group showed a significantly higher eating difficulty in meats, compared to the YA group (Mann–Whitney *U* test, two‐tailed *p* = 0.017). The findings support Hypothesis 2 that the use of adaptive/maladaptive strategies was associated with aging.

**FIGURE 3:**
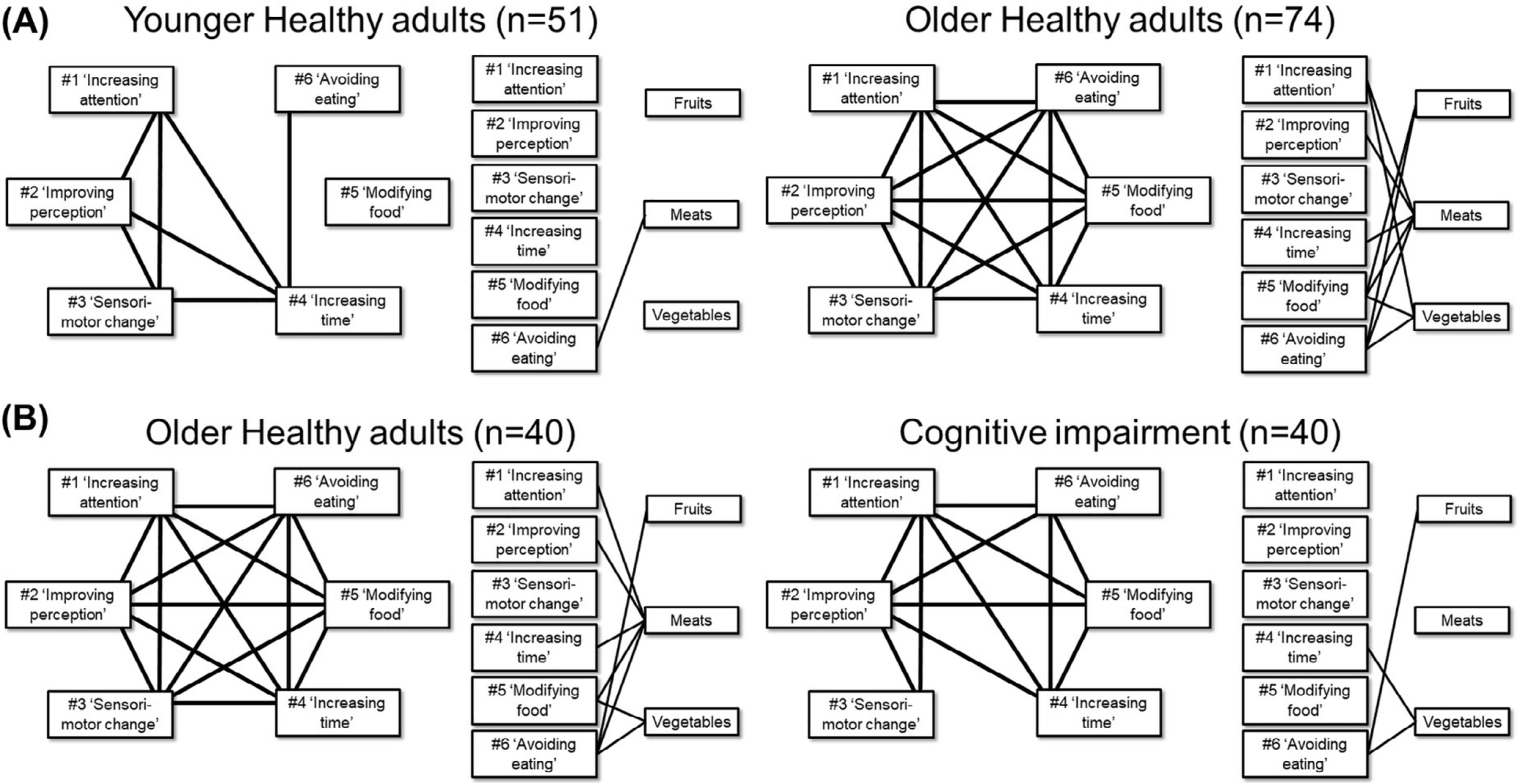
The pattern of cross‐correlation among the adaptive/maladaptive strategies in each group. The solid line denotes a correlation between the scores of two strategies with statistical significance. (A) The comparison between younger healthy adults and older healthy adults (from Study Set 1). (B) The comparison between the patients with cognitive impairment (from Study Set 2) and a subgroup of age and sex matched older healthy adults.

As shown in Figure 3A, in the OA group, the scores from all the six strategies were significantly cross‐correlated, suggesting that individuals who used one of the strategies would concurrently use other strategies. In contrast, the YA group showed a significant cross‐correlation only within the first four strategies (all adaptive strategies). The use of adaptive strategy #5 ‘Modifying food’ and the maladaptive strategy #6 ‘Avoiding eating’ were mostly dissociated from the other four (Figure 3A). The pattern of the association between strategies and eating difficulty in different food types also differed between age groups. As shown in Figure 3A, the OA group was likely to use all the strategies (except for #3) when there was greater eating difficulty for all food types. In contrast, the YA group only used the maladaptive strategy #6 when there was difficulty eating meats. The findings suggested a substantial difference in the pattern of using the strategies between the age groups.

### 3.4 Results of Hypothesis 3—Cognitive Impairment and Masticatory Adaptation

The comparison between CI patients and the YA group did not show a significant difference in the MAEQ mean score and the score from each of the strategies or foods, except that the CI patients were more frequently using the maladaptive strategy #6 ‘Avoiding eating (Mann–Whitney *U* test two‐tailed *p* < 0.001). Compared to the YA group, the CI patients showed a greater degree of eating difficulty for all foods (Mann–Whitney *U* test two‐tailed *p* = 0.049) and for fruits (Mann–Whitney *U* test two‐tailed *p* = 0.016).

The comparison between CI patients and a subset of the OA group (matched for age and sex) revealed no significant difference between the MAEQ mean score or the score for each strategy. The difference between eating difficulty, as reported in a previous study [28], was not statistically significant. The findings failed to support Hypothesis 3 that patients with CI showed a higher MAEQ score.

In contrast, the analysis of cross‐correlation showed a substantial difference between the OA group (matched for age and sex) and the CI patients. As shown in Figure 3B, the OA group used multiple strategies when having greater difficulty. In contrast, in CI patients, the association between the use of the strategies and eating difficulty was mostly dissociated, with the exceptions of avoiding eating fruits or vegetables and increasing time to eat vegetables (Figure 3B).

### 3.5 Predictive Models of Eating Difficulty

In the OA group (*n* = 74), the regression analysis of the MAEQ mean score showed that only three variables reached a statistical significance: the number of missing teeth (*β* = 0.34, *p* = 0.017), masticatory performance (*β* = 0.28, *p* = 0.039) and the MAEQ mean score (*β* = −0.23, *p* = 0.025) were the factors predicting eating difficulty with statistical significance, with a DW statistic = 1.98 and all VIFs < 4. The regression analysis of multiple strategies revealed two predictors with statistical significance, that is, the number of missing teeth (*β* = 0.31, *p* = 0.010) and the score from MAEQ #6 (*β* = −0.38, *p* = 0.016), with a DW statistic = 1.88 and all VIFs < 5. All the findings showed a lower risk of multicollinearity and autocorrelation.

In contrast to the OA group, in the CI patients, the regression analysis of the MAEQ mean score showed that none of the predictors was statistically significant. The regression analysis of multiple strategies revealed that only the score from MAEQ #6 was the predictor with statistical significance (*β* = −0.53, *p* = 0.012), with a DW statistic = 1.43 and all VIFs < 4. The findings suggest that in older individuals with and without CI, a higher frequency of avoiding eating was associated with a higher eating difficulty in all food types.

## 4 Discussion

### 4.1 Major Findings From the Study

In this study, the MAEQ is developed to assess the frequency of using adaptive/maladaptive strategies of mastication in older individuals. Notably, the MAEQ has not been validated in patients with severe dementia or those who have difficulties with language/comprehension. Therefore, the current MAEQ should be used only in individuals with no or mild‐to‐moderate CI. The current findings revealed that (a) the MAEQ showed good reliability and content validity. However, good criterion‐related validity was only found in the OA group. (b) Increased age may be a critical factor associated with the higher frequency of using masticatory adaptive strategies. (c) The OA groups used multiple strategies concurrently when there was great eating difficulty. In contrast, CI patients showed a weak association between the use of adaptive strategies and eating difficulty. (d) Both the OA groups and CI patients showed a higher frequency of avoiding eating (i.e., a maladaptive strategy) when eating difficulty was great.

### 4.2 The Use of Adaptive/Maladaptive Strategies for Eating Difficulty

As shown in Figure 1, Strategy #3 ‘Sensorimotor change’, such as changing the other side to chew, was the most frequently used strategy for both the YA and the OA groups. This strategy requires tongue movement to transport the food bolus, and sensory feedback plays a key role in the sensorimotor control of the oral apparatus. For example, clinical research revealed that in older individuals, better oral diadochokinesis, which requires fine control of tongue movement, was associated with better masticatory performance [32]. Animal research revealed that deficits in oral sensation impaired the coordination of tongue and jaw movements [33]. Nevertheless, in the OA group, individuals showing greater difficulty eating daily meals did not show an increased use of Strategy #3, as demonstrated by the lack of significant correlation between the two factors (Figure 3A).

In contrast to the adaptive strategies, in the OA group, greater eating difficulty was associated with an increased use of the maladaptive strategy #6 ‘Avoiding eating’ (see Section 3.5). The findings suggest that in older individuals, while a variety of adaptive strategies were frequently used to maintain eating, avoidance of food intake was more common when there was greater difficulty in eating daily meals. Food avoidance has been widely reported in previous studies, especially for patients with poor oral conditions (e.g., multiple tooth loss) [34, 35]. The current results provide additional evidence that eating difficulty was not only associated with oral health (i.e., an increased number of missing teeth) but also with the use of the maladaptive strategy (see Section 3.5).

### 4.3 Age‐Related Factors Play a Key Role in Masticatory Adaptation

In contrast to the OA group, the YA group did not show a significant correlation between the MAEQ mean score and eating difficulty. The finding may be interpreted by the distribution of the score of eating difficulty, which was substantially right‐tailed (with the majority of them showing no eating difficulty). The results correspond to previous findings that younger individuals more likely preferred rough and hard foods, compared to older individuals [35]. In the YA group, although the lack of significant correlation between the MAEQ scores and eating difficulty (see Section 3.2) failed to support a criterion‐related validity, a previous study revealed that TMD patients—which were mostly younger individuals—showed a lower MAEQ score (i.e., more use of adaptive/maladaptive strategies) than healthy participants [31]. The findings suggest that the MAEQ score can discriminate between a healthy and a suboptimal condition of mastication.

A key finding from the current study is that the OA group and the CI group did not significantly differ in their MAEQ scores, which disconfirmed Hypothesis 3. An interpretation of this finding is that CI patients, though having difficulty in eating and worse oral health [36], have a strong tendency to cope with the conditions and try to use different adaptive/maladaptive strategies, as the OA group did. However, they may not successfully cope with these conditions, judging from a dissociation between the use of strategies and eating difficulty (Figure 3B). The analyses of cross‐correlation revealed the difference between the OA group and the CI patients: both of them showed an increased use of the maladaptive strategy (i.e., #6 ‘Avoiding eating’) as well as greater eating difficulty, compared to the YA group. However, while the OA group adopted multiple adaptive strategies of mastication when eating difficulty was high (Figure 3B), in CI patients, the association between using adaptive strategies and eating difficulty was weak (Figure 3B). The findings suggest a ‘dissociation’ between using adaptive strategies and increasing eating difficulty in CI patients. The finding suggested that cognitive impairment may play a key role in two aspects of food intake. In CI patients, even when these strategies were used, the frequencies of using them were weakly correlated with their eating difficulty. The findings suggest that in CI patients, the use of adaptive strategies may not successfully reduce their difficulty in eating daily meals.

### 4.4 Limitations and Further Considerations

The findings from this study should be interpreted cautiously with the following considerations. First, as a cross‐sectional study, the findings should not be interpreted as the causal links between masticatory adaptation and eating difficulty. On the one hand, longitudinal research showed that a poor masticatory ability to chew hard food was a risk factor for malnutrition [4]. On the other hand, malnutrition was a predictive factor associated with a higher risk of cognitive decline [37], which impaired one's ability to use adaptive behaviours.

Second, we did not exclude the potential effect of oral and systemic conditions on masticatory adaptation and eating in CI patients. In Study Set 2, the collection of clinical variables (e.g., the number of missing teeth) was limited because the study was conducted during the COVID‐19 pandemic. Nevertheless, judging from the findings from the literature, it is reasonable to conceive that poor oral health, as commonly seen in CI patients [36], plays a key role in their masticatory adaptation. Consistently, systemic diseases (e.g., gastrointestinal or salivary gland diseases) may influence patients' food selection and eating experience.

Third, we assessed eating difficulty using a food‐specific questionnaire. The approach reflects the difficulty for individuals to eat daily meals. The food‐specific approach increases the clinical significance of the results because the foods consumed in a daily meal are highly regionally and culturally specific [15, 16]. However, our findings are based on Asian food (mainly Taiwanese cuisine), and the results should be carefully generalised to individuals from other regions and cultures.

### 4.5 Clinical Implications

The study presents novel findings on the association between the subjective experience of masticatory adaptation and the difficulty of eating daily meals. While previous studies focused on quantifying the masticatory performance or experience of eating, the current findings highlight that behavioural adaptation—how individuals cope with their challenges in eating meals—plays a significant role in food intake. Notably, the MAEQ presented here was not used for screening ‘good vs. bad chewers’. Instead, it may help clinicians and healthcare workers to spot what strategies (or combination of them) individuals used in coping with their masticatory difficulty, and form an individualised care plan to improve their food intake. Clinicians and healthcare workers should pay attention to the maladaptive behaviour of mastication, that is, avoiding eating foods, in older individuals. While older individuals without CI can adopt a variety of adaptive strategies, the CI patients mainly avoid eating foods. As a behaviour of diet choice and eating habits, avoiding eating some foods is considered a behavioural adaptation in individuals with masticatory dysfunction [11]. However, this behaviour may cause a long‐term negative outcome to one's nutritional status. Such a behaviour of masticatory adaptation should be critically monitored for the CI patients.

In terms of using the MAEQ for clinical assessment, one should keep in mind that the CI patients in the current study were screened for their ability to communicate verbally. Therefore, the validity of the MAEQ should be very carefully generalised to patients with other deficits of CI. For example, patients in late‐stage dementia have severe deficits in language and comprehension, and therefore the assessment based on questionnaires can be invalid, and the data collected could be misleading. For patients with CI, the MAEQ should be conducted only when patients are evaluated by a neurologist for their cognitive ability to comprehend the meaning of the descriptions.

## 5 Conclusion

The MAEQ is a valid tool for assessing the use of adaptive/maladaptive strategies during mastication in older individuals. Aging and cognitive impairment may play a key role in how individuals use the strategies.

## Author Contributions

**Chia‐Shu Lin:** Writing – review and editing, writing – original draft, methodology, investigation, formal analysis, data curation, conceptualisation. **Ta‐Chung Chen:** Writing – review and editing, methodology, data curation, conceptualisation. **Yi‐Chen Chen, Li‐Jung Chao, and Wei‐Chieh Kao:** Methodology, Investigation. **Jong‐Ling Fuh:** Writing – review and editing, methodology, data curation, conceptualisation.

## Ethics Statement

The participants from Study Set 1 and Set 2 have signed a written informed consent before the research began. Study Set 1 was approved by the Institutional Review Board (IRB) of the NYUC (Code: YM109057F), and Study Set 2 was approved by the IRB of the VGHTPE (Code: 2021‐08‐004CC).

## Consent

All the participants completed the written informed consent before the study started.

## Conflicts of Interest

The authors declare no conflicts of interest.

## Supporting information


**Table S1:** The Chinese version of Masticatory Adaptation Experience Questionnaire.

## Data Availability

The data that support the findings of this study are available from the corresponding author upon reasonable request.
