# Supplementary material for: Assessing the Adaptive/Maladaptive Strategies of Mastication in Older Taiwanese Individuals Using the Masticatory Adaptation Experience Questionnaire (MAEQ)
Source: J Oral Rehabil. 2026 Mar 10;53(7):1306–18. doi: 10.1111/joor.70181 (PMC13261772; doi:10.1111/joor.70181)
Supplement: Supplementary file 1 — Table S1: The Chinese version of Masticatory Adaptation Experience Questionnaire. [file JOOR-53-1306-s001.pdf]

咀嚼適應經驗問卷
(Masticatory Adaptative Experience Questionnaire (MAEQ)

請回答當您在**<u>過去三個月內</u>**進食以下四種食物時，多常有下列的經驗。

請詳細閱讀下列描述並以勾選適合的數字。數字代表**<u>有多常發生這種經驗</u>**。

1<br>總是	2<br>經常	3<br>偶爾	4<br>很少	5<br>從不

當我吃<u>柳丁(切片處理)</u>時

1	我會特別注意牙齒(或假牙)是否把它咬爛或咬碎。	1	2	3	4	5	
2	我會特別去感覺食物(食團)在口中的大小、軟硬與質地(例如彈性與黏稠感)。	1	2	3	4	5	
3	我會用不同邊或不同部位的牙齒，或是用口腔其他部位(例如牙床)，來咬爛或咬碎它。	1	2	3	4	5	
4	我會試著花更多時間或是更用力去嚼它。	1	2	3	4	5	
5	我會因為不太容易吃這種食物，而改變烹調或處理它的方式(例如蒸煮或切小塊再吃)。	1	2	3	4	5	
6	我會避免吃這種食物，因為對我而言很難咬爛或咬碎它。	1	2	3	4	5	

當我吃<u>芭樂(切片處理)</u>時

1	我會特別注意牙齒(或假牙)是否把它咬爛或咬碎。	1	2	3	4	5	
2	我會特別去感覺食物(食團)在口中的大小、軟硬與質地(例如彈性與黏稠感)。	1	2	3	4	5	
3	我會用不同邊或不同部位的牙齒，或是用口腔其他部位(例如牙床)，來咬爛或咬碎它。	1	2	3	4	5	
4	我會試著花更多時間或是更用力去嚼它。	1	2	3	4	5	
5	我會因為不太容易吃這種食物，而改變烹調或處理它的方式(例如蒸煮或切小塊再吃)。	1	2	3	4	5	
6	我會避免吃這種食物，因為對我而言很難咬爛或咬碎它。	1	2	3	4	5	

版本：2.0 2021.12.4

請注意背面還有題目

請回答當您在**過去三個月內**進食以下四種食物時，多常有下列的經驗。

請詳細閱讀下列描述並以勾選適合的數字。數字代表**有多常發生這種經驗**。

1<br>總是	2<br>經常	3<br>偶爾	4<br>很少	5<br>從不

當我吃炒花生時

1	我會特別注意牙齒(或假牙)是否把它咬爛或咬碎。	1	2	3	4	5	
2	我會特別去感覺食物(食團)在口中的大小、軟硬與質地(例如彈性與黏稠感)。	1	2	3	4	5	
3	我會用不同邊或不同部位的牙齒，或是用口腔其他部位(例如牙床)，來咬爛或咬碎它。	1	2	3	4	5	
4	我會試著花更多時間或是更用力去嚼它。	1	2	3	4	5	
5	我會因為不太容易吃這種食物，而改變烹調或處理它的方式(例如蒸煮或切小塊再吃)。	1	2	3	4	5	
6	我會避免吃這種食物，因為對我而言很難咬爛或咬碎它。	1	2	3	4	5	

當我吃炸雞腿、雞排時

1	我會特別注意牙齒(或假牙)是否把它咬爛或咬碎。	1	2	3	4	5	
2	我會特別去感覺食物(食團)在口中的大小、軟硬與質地(例如彈性與黏稠感)。	1	2	3	4	5	
3	我會用不同邊或不同部位的牙齒，或是用口腔其他部位(例如牙床)，來咬爛或咬碎它。	1	2	3	4	5	
4	我會試著花更多時間或是更用力去嚼它。	1	2	3	4	5	
5	我會因為不太容易吃這種食物，而改變烹調或處理它的方式(例如蒸煮或切小塊再吃)。	1	2	3	4	5	
6	我會避免吃這種食物，因為對我而言很難咬爛或咬碎它。	1	2	3	4	5	
